# A Global PPR Network for Field Staff

**DOI:** 10.3389/fvets.2019.00267

**Published:** 2019-08-20

**Authors:** Paul Rossiter

**Affiliations:** St. Michael's House, Devon, United Kingdom

**Keywords:** peste des petits ruminants, information sharing network, field staff, motivation, epidemiological evidence gathering, disease eradication

## Introduction

Among the lessons learned from rinderpest eradication was that networks developed for laboratory testing for diagnosis and for serological assays for rinderpest virus antibody proved very valuable to the final success of the Global Rinderpest Eradication Programme. The Joint Division of the Food and Agriculture Organization of the United Nations (FAO) and the International Atomic Energy Agency (IAEA) co-ordinated perhaps the best known of these networks across Africa. This network successfully built and supported a team of skilled laboratory staff across the continent who knew, trusted and collaborated with one another, and from which many individuals progressed to important positions in national, regional, and global animal disease control.

The value of such networks for PPR control and eradication, as well as collaboration between laboratories in general, encouraged two meetings of the Global PPR Research Alliance (GPRA) after which the FAO and the World Organization for Animal Health (OIE) established a Global Research and Expertise Network (GREN) as an integral part of the PPR-Global Eradication Programme. The background and potential of GREN was explored and developed during an electronic conference held in February to April 2014 and GREN was launched at the IAEA headquarters in Vienna in April 2018.

The author contributed to the first meeting of the GPRA, and subsequently, as moderator of the e-conference in 2014, had first-hand access to individual inputs and ideas and drafted the final report. He presented a summary of these and additional findings at the launch of GREN in 2018. This paper re-caps some of the findings and concepts in those presentations and reports. It then makes the case that whilst GREN should actively encourage and develop technical networking at the laboratory level it should not miss the opportunity to establish an equivalent programme for staff involved in field operations.

## Early Steps: 1—The Global PPR Research Alliance, 2012–2013

Following the official confirmation of global rinderpest eradication in 2011, the Pirbright Institute and others established a forum, GPRA, for greater collaboration amongst scientists studying PPR. The GPRA met twice, in London and in Nairobi. The outcomes were clear; substantial research was being carried out on PPR, much of this was at a sophisticated technical level, and there was perhaps insufficient collaboration and study in the field. The author gave a brief presentation attempting to highlight the advantages that might come from more involvement with and coordination of field workers (1). The suggested potential benefits included a better understand of local patterns of disease (endemic areas, main factors behind spread of infection, seasonality, the dynamics of herd structure, and recruitment of susceptibles, etc.) and the chance to maximize efforts for better control (best opportunities to interrupt chains of virus transmission, minimum immunity rates to break transmission, use of different vaccines), and defining the true role of other susceptible species in the overall epidemiology of PPR. Several of these issues have or are now being addressed by established research teams (2–5) but the opportunity for more observational and practical research in the field is still there, as is the potential for increased awareness and willingness to search for and report possible cases of PPR.

### The Take-Away Message

There is considerable human interest and potential for studying PPR in the field as well as the laboratory.

## Early Steps: 2—The GREN e-conference, 2014

### Participation

The moderated, part-time e-conference was held over a period of 5 weeks in early 2014. An immediate difference to the earlier rinderpest eradication research networking, was that GREN would not work just with laboratory-based scientists but would encourage policy and operational level staff to contribute their expertise into the discussions. Over 300 participants registered with 90 making at least one contribution. There was a strong laboratory-based bias to participants ranging from internationally recognized scientists in the accredited laboratories, to national labs through to aspiring researchers and their students in small veterinary colleges and provincial diagnostic laboratories. However, there were also many participants who were not laboratory-based and who were working in administrative and operational positions in national and regional programmes, including a considerable number contributing in the field through vaccine delivery and disease investigation, several of whom were newly qualified veterinarians. Geographic participation covered most countries where PPR was a problem, as well as some where PPR is yet to occur and may never occur. The potential for the e-conference was under-realized with 40 would be participants asking to be registered on the final day of the conference, and individual requests to join the conference still being received as recently as July 2019.

### Ideas and Concepts From the e-conference

The proposed purpose of the GREN was “to accelerate the progressive control of PPR through a forum that distributes new knowledge about the virus, the disease, and improved methods for its control.” The conference concluded that the concept of GREN as a forum for information exchange was (and still is) very welcome. Whilst it was not the purpose of the e-conference to decide exactly what format the GREN should follow—it did propose that a moderated global network with an open system for reports and queries and possible monthly structured “seminars” and question times would help keep the momentum for eradication going. In view of language issues and regional programmes, it might also want to consider global, regional, and national components.

The e-conference was successful and well-received by most participants. One shortcoming however was that the rigid framework provided by the organizers focused predominantly on scientific and technical subjects with the result that crucial issues such as motivation, resourcing, promotion, and general awareness were not discussed or only briefly.

### The Take-Away Message

It was impossible to ignore the enthusiasm and interplay between participants with different backgrounds and expertise: the willingness of several internationally acclaimed scientists to engage with and answer questions from recent graduates was noticeably positive. Many of the questions being asked were very perceptive and open requiring careful answers, and a few were completely off the wall, which proved equally challenging and stimulating. This ability of the GREN to foster exchange of ideas between senior scientists and inexperienced field and lab staff was a powerful example of how the “experience” component of GREN might work and could be a strong source of motivation for staff joining the PPR eradication community (6).

## The Launch of GREN, 2018

PPR-GEP GREN was officially launched in April 2018 at a meeting in Vienna. The presentations at the meeting were predominantly institutional and laboratory-science focused. However, the author was given the opportunity to recount his experience with the e-conference and argued for a wider base for GREN: “Whilst the inaugural emphasis is largely laboratory-based the network should aim to include field workers and programme managers who, by reporting what they are experiencing in the field, including clinical signs and pathology, and their successes and difficulties in achieving control, will stimulate discussion and sharing of the best methods for disease surveillance and immunization. By harnessing and guiding this widespread enthusiasm to share knowledge about PPR, GREN can be the tool to effectively promote and harmonize the global effort against the virus making the 2030 target for its elimination more feasible” (7).

The final communique of the meeting (8) presented the Terms of Reference for GREN which includes nine specific roles and functions of the network. First on this list is that the GREN shall “Serve as a communication and technology sharing gateway for the PPR GEP to coordinate inclusive field collaboration across the PPR-GEP community.” If the gate to the gateway is permanently open, operating in both directions to allow coordination and knowledge of new technology to pass outwards, and information from the field to flow inwards—this could be a very powerful tool for PPR-GEP management and for gathering epidemiological information.

### The Take-Away Message

The GREN intends to promote field collaboration across the PPR-Community.

## Discussion and Conclusion

As with rinderpest, the PPR-GEP strategy will concentrate on eliminating clinical PPR followed by intensive clinical and serological surveillance to confirm eradication of infection with the virus. The main source of evidence to support clinical elimination is the field through routine reports, outbreak reports, participatory epidemiology, specific targeted disease searching, etc. Unfortunately, these channels are not always used to their maximum advantage but might be more effective if the field staff generating the information was more highly motivated. Other than a massive pay rise, what might induce such motivation? The three take-away messages from the sections above combine to strongly suggest that a network as outlined will be well-received and likely to increase field workers sense of inclusion in the overall global eradication process. The 2014 GREN e-conference showed a clear desire for knowledge about PPR at all levels of participation including the field front-line. Publishing all PPR-GEP newsletters and appropriate reports on the GREN would be of widespread interest to the PPR community. Many field staff would be interested in new developments in PPR control but, unlike staff in most established laboratories, do not have easy access to new reports and papers. PPR-GEP could consider using GREN to disseminate the OIE's “PPR Watch” list of recent PPR publications to the wider PPR community. Providing this monthly list to all members of a field-based GREN would be much appreciated—especially if donor funds could ensure that there was open access to all papers for GREN members. Such inputs could be easily repaid in terms of information flowing back from the field. A network where front line staff from Bulgaria to Bangladesh and Burundi, from Tunisia to Tajikistan and Tanzania could share their ideas and descriptions of disease, share their photos of clinical signs between themselves and with PPR-GEP would allow them to feel more involved, especially where their efforts are appreciated and acknowledged, and possibly acted upon. Raising staff interest, moral, and sense of participation in the global programme should increase disease reporting, feedback about control programmes, and early warning of problems and difficulties. With virtually all staff now possessing powerful smart phones the technology is already available in the field, all that is required is the network, and its coordination and management. Make a brief field visit anywhere in the world where there is plenty of PPR and you will probably be shown more good photos of the clinical signs and even the pathology of PPR than exist in any textbook or training manual. The same is also true of what is known about the epidemiology of PPR at local levels and of what “works best” to stop the disease in such locations. How useful it would be to share and discuss this untapped experience. If greater motivation in the field could hasten the global eradication of PPR by just one year, how much time, resources, and funding might be saved?

The details of how such a network should be run will require further deliberation, checks, and balances to ensure that information in the network does not by-pass national and regional programmes and reporting structures. Therefore, the proposal initially concentrates on veterinary front-line staff in the public or private sector under the overall direction of the DVS but in the field there are many more actors and stakeholders who can contribute to disease reporting. These include community animal health workers, local government, and NGO service providers, as well as farmers and herders themselves. Many of these, especially herders, greatly outnumber front-line field staff which could make receiving, assessing, and responding to information from them a time-consuming job requiring the possible recruitment of extra staff to cope with this workload. Consequently, it might be practical to begin the field network with veterinary front-line staff alone in order to keep it manageable. If successful, the next step could be to enlarge the network at country level incorporating other actors and methodologies deemed appropriate in that location—and ensuring the flow of information is always accessible to the national veterinary services.

The proposed network could also provide other opportunities for GREN. In addition to collaboration in the field, the final communique of the inaugural meeting of GREN highlighted closer interaction between epidemiology and socio-economics as a “thematic area” and a “Priority Research Need.” The field network could be a valuable entry point for socio-economic input. The involvement of socio-economists in the development and management of the network would increase its value especially where this leads to better understanding of some of the constraints to disease reporting and how these can be removed to increase the flow of useful epidemiological information. In addition, socio-economic involvement from the outset could help to provide the data to show, at a later date when examining the progress of PPR-GEP, whether field networking actually improves disease reporting, resulting in earlier eradication of PPR and consequent economic savings. If the proposed network could be shown to do this, it would significantly help PPR-GEP GREN “to accelerate the progressive control of PPR.”

## Author Contributions

The author confirms being the sole contributor of this work and has approved it for publication.

### Conflict of Interest Statement

The author declares that the research was conducted in the absence of any commercial or financial relationships that could be construed as a potential conflict of interest.
